# Editorial: The effects of food processing on food components and their health functions, Volume II

**DOI:** 10.3389/fnut.2022.1051869

**Published:** 2022-11-07

**Authors:** Jinkai Zheng, Hang Xiao

**Affiliations:** ^1^Institute of Food Science and Technology, Chinese Academy of Agricultural Sciences, Beijing, China; ^2^College of Food Science and Engineering, Qingdao Agricultural University, Qingdao, China; ^3^Department of Food Science, University of Massachusetts, Amherst, MA, United States

**Keywords:** food, components, processing, properties, health functions

Food processing plays an important role in our food supply and can significantly influence the properties of food components and their impact on health (1, 2). This Research Topic is focused on the effects of food processing on food components, and their physicochemical properties and health functions (Figure 1). The studies demonstrated that various processing methods (e.g., drying, extraction, ultra-high pressure treatment, hydroxylation, backing, and dehydration) significantly influenced the non-volatile and volatile chemical components in foods, which further affected their health-promoting effects. Processing parameters such as temperature, enzymes, pressure, and treatment time are also important and should be considered carefully.

**Figure 1:**
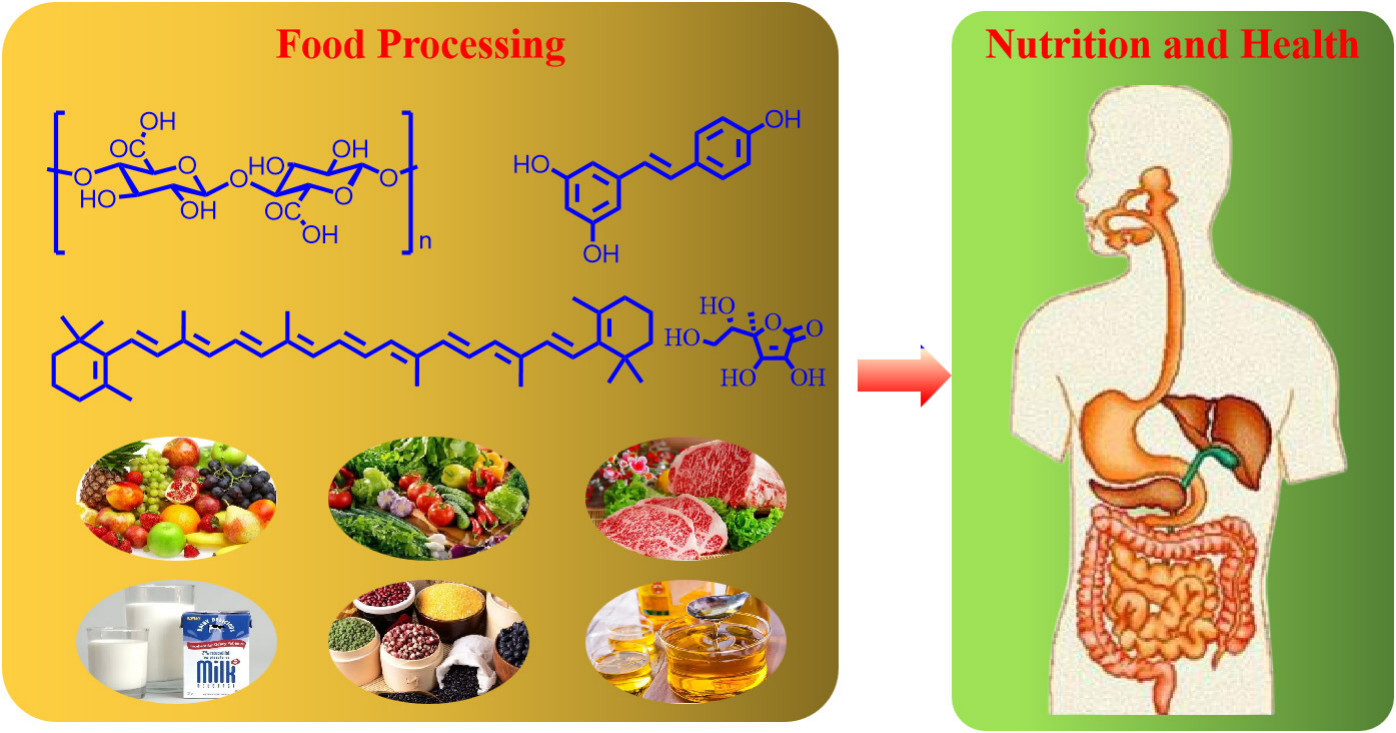
Food processing affecting food components and their health functions.

Liu, Meng, et al. investigated the transformation of non-volatile taste components in Stropharia rugosoannulata subjected to hot air drying, vacuum freeze drying, and microwave vacuum drying. The diversity of metabolites in the samples changed little in response to the three drying methods tested, but their relative abundance and metabolic pathways differed significantly. This study also showed that the metabolite differences caused by different drying methods are modulated by temperature (higher temperatures may cause protein degradation), Strecker degradation, and the Maillard reaction. Liu, Mao, et al. also showed that high pressure treatment significantly increased the ordered structure content such as α-helices, inhibited the formation of disulfide bonds, and decreased the surface hydrophobicity of myofibrillar proteins (MP). Moreover, MP had the optimal solubility and *in vitro* digestibility at 200 MPa due to the minimum particle size and turbidity, and relatively dense and uniform microstructure. The ultrahigh pressure treatment effectively improved the digestibility of MP from scallop mantle. Processing facilitated the inter-conversion of components, and components with high value could be obtained by modulating the processing parameters. Li, Shao, et al. studied the effects of amino acids and processing methods on the conversion of ginsenosides in American ginseng into rare ginsenosides. The results showed that aspartic acid was the best catalyst, and thermal extraction had the best effect. Qiao, et al. obtained a novel angiotensin I-converting enzyme inhibitory peptide by hydrolyzing goat milk casein. Feng, et al. developed two-step vacuum-mediated conversion from fatty acid ethyl esters and fatty acids into a lipid-lowering diacylglycerol-enriched functional oil derived from soy sauce by-product oil. Lipase loading, temperature, substrate molar ratio, and initial vacuum combination significantly affected the reaction efficiency. Wang, Nie, et al. demonstrated that the flavonoid, phenolic acid, γ-GABA, and polysaccharide contents and antioxidant activity during the germination of buckwheat gradually trended upward, and the antioxidant properties of buckwheat sprouts might be related to the phenolic acid and polysaccharide contents, which were improved by plasma-activated water treatment.

In addition to non-volatiles, significant variation in volatiles was detected during processing. Wang, Yu, et al. characterized the transformation and formation of volatile metabolites during green tea processing. They showed that volatile compounds underwent different changes during various procedures, with the greatest transformation occurring during fixation, followed by pan-frying and second drying. During the fixation process, high temperature promoted the degradation of lipids to generate free fatty acids, which led to the formation of volatile alcohols and aldehydes. Glycosides were also degraded into multiple volatiles. Baking significantly affected the chemical composition, sensory quality, and bioactivity of Tie Guan Yin Oolong Tea (Gao, et al.). Baking promoted the formation of colored macromolecules (e.g., theabrownins), which affected the color of the tea infusion. Floral and fresh volatiles were remarkably reduced, while multiple new volatiles were produced, resulting in a typical baked aroma. The antioxidant and antibacterial activities were reduced after baking, which might be associated with the decrease in monomeric catechins. Dehydration increased the food shelf life and affected the composition and physicochemical properties of food. For example, most aldehydes and alcohols were increased when Pugionium is subject to different dehydration procedures, whereas esters were decreased, and the dehydrated Pugionium has a more harmonious, less pungent aroma compared with the fresh Pugionium (Li, Wu, et al.).

In summary, processing significantly affects food properties, especially their chemical compositions, physicochemical properties, and health effects. Targeted modulation of foods could be achieved by regulating processing. Due to their nutritional and health properties, the development and application of functional foods is attracting increasing attention. Since individuals respond differently to the same foods and their bioactive compounds (3), precision nutrition is regarded as a promising strategy for maintaining human health and preventing disease. Precise targets of foods based on the systematic study of the health-promoting effects of various foods and their components is the basis for precision nutrition. Precise manufacture of foods based on the development of novel techniques is a critical part of precision nutrition. Precise detection techniques for real-time dynamic monitoring of *in vivo* biomarkers is also important to the realization of precision nutrition (4). Notably, traditional precision nutrition emphasizes the final function of food. However, foods are a complex mixture of components that interact with each other and influence the health-promoting effects of foods generally. There is complex variation in food chemical compositions during processing, as well as in the human body (3, 5). Therefore, to achieve precise nutrition, the molecular targets of food components need to be identified and the food composition needs to be precisely regulated. Such information would provide critical scientific basis for the design and manufacture of innovative health-promoting foods, which in turn promote the realization of precision nutrition.

## Author contributions

JZ and HX prepared, checked and revised the manuscript, and approved the submitted version.

## Conflict of interest

The authors declare that the research was conducted in the absence of any commercial or financial relationships that could be construed as a potential conflict of interest.

## Publisher's note

All claims expressed in this article are solely those of the authors and do not necessarily represent those of their affiliated organizations, or those of the publisher, the editors and the reviewers. Any product that may be evaluated in this article, or claim that may be made by its manufacturer, is not guaranteed or endorsed by the publisher.
